# Supplementary material for: Atomic structure of the innexin-6 gap junction channel determined by cryo-EM
Source: Nat Commun. 2016 Dec 1;7:13681. doi: 10.1038/ncomms13681 (PMC5146279; doi:10.1038/ncomms13681)
Supplement: Supplementary Information — Supplementary Figures 1-8, Supplementary Table 1 and Supplementary References. [file ncomms13681-s1.pdf]

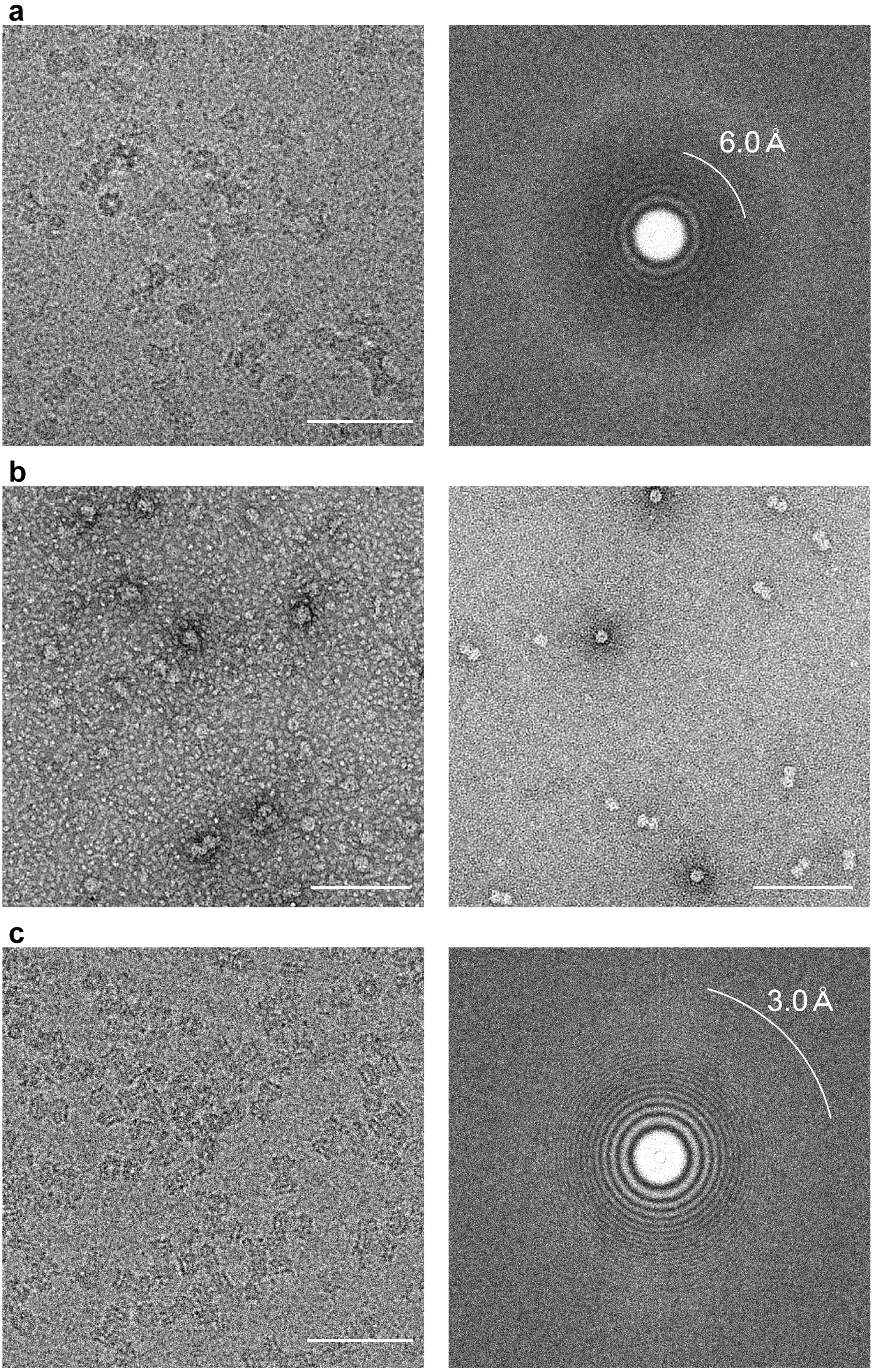
a
6.0 Å
b
c
3.0 Å

Supplementary Figure 1 | Electron microscopy for purified INX-6 channels. a, (left) Representative cryo-electron micrograph of INX-6 channels in 0.1% OGNG detergent solution. (right) The corresponding fast Fourier transform image shows Thon rings extending to only ~6 Å resolution. Scale bar, 50 nm. **b,** Negatively-stained electron micrographs of INX-6 channels before (left) and after (right) GraDeR[1]. Whereas a lot of LMNG micelles are observed in the background before GraDeR (left), the grid after GraDeR shows a clean background (right). Scale bars, 100 nm. **c,** (left) Representative cryo-electron micrograph of INX-6 channels after GraDeR. (right) The corresponding fast Fourier transform image shows Thon rings extending to 3 Å resolution. Scale bar, 50 nm.

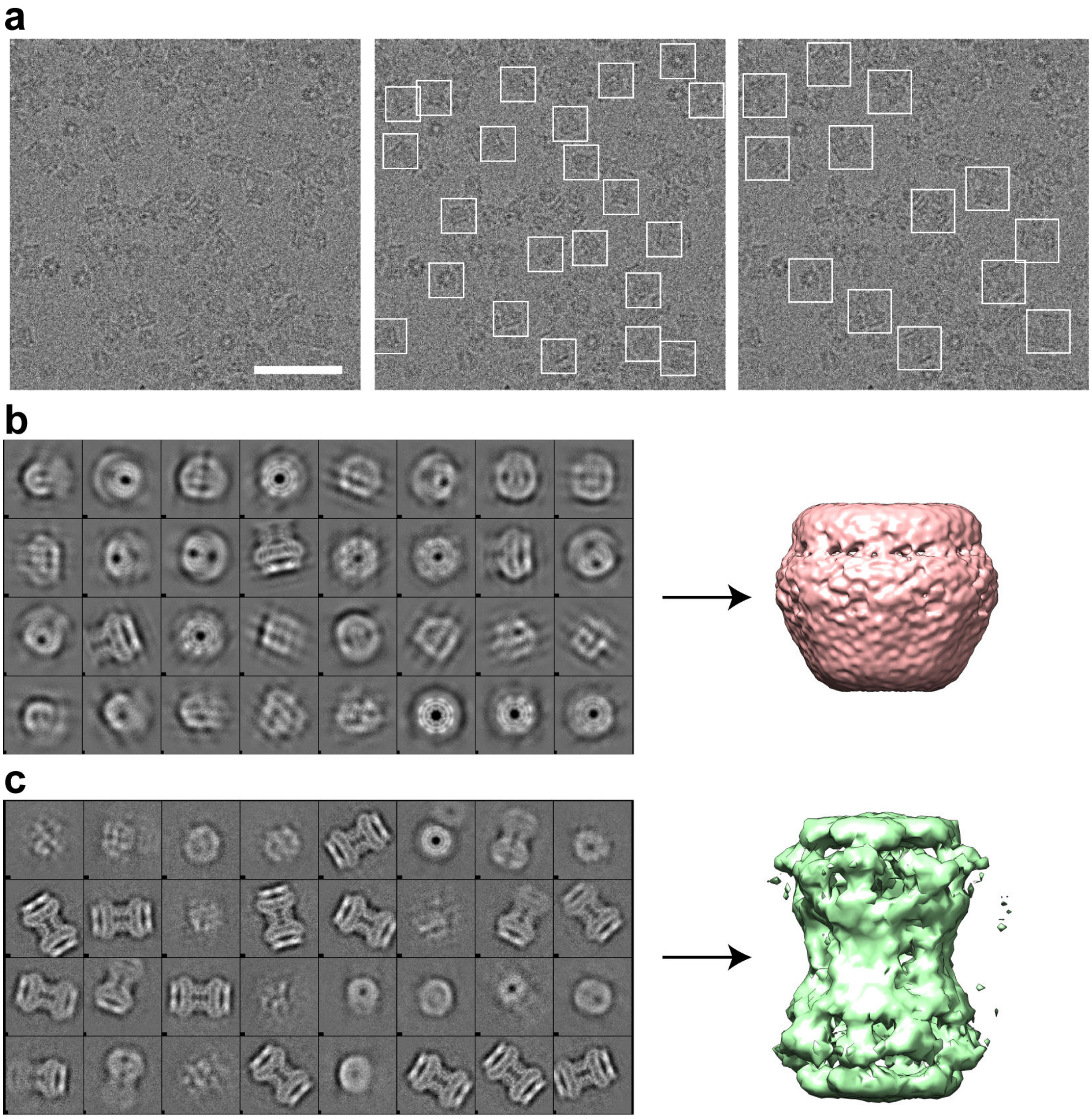
a
b
c

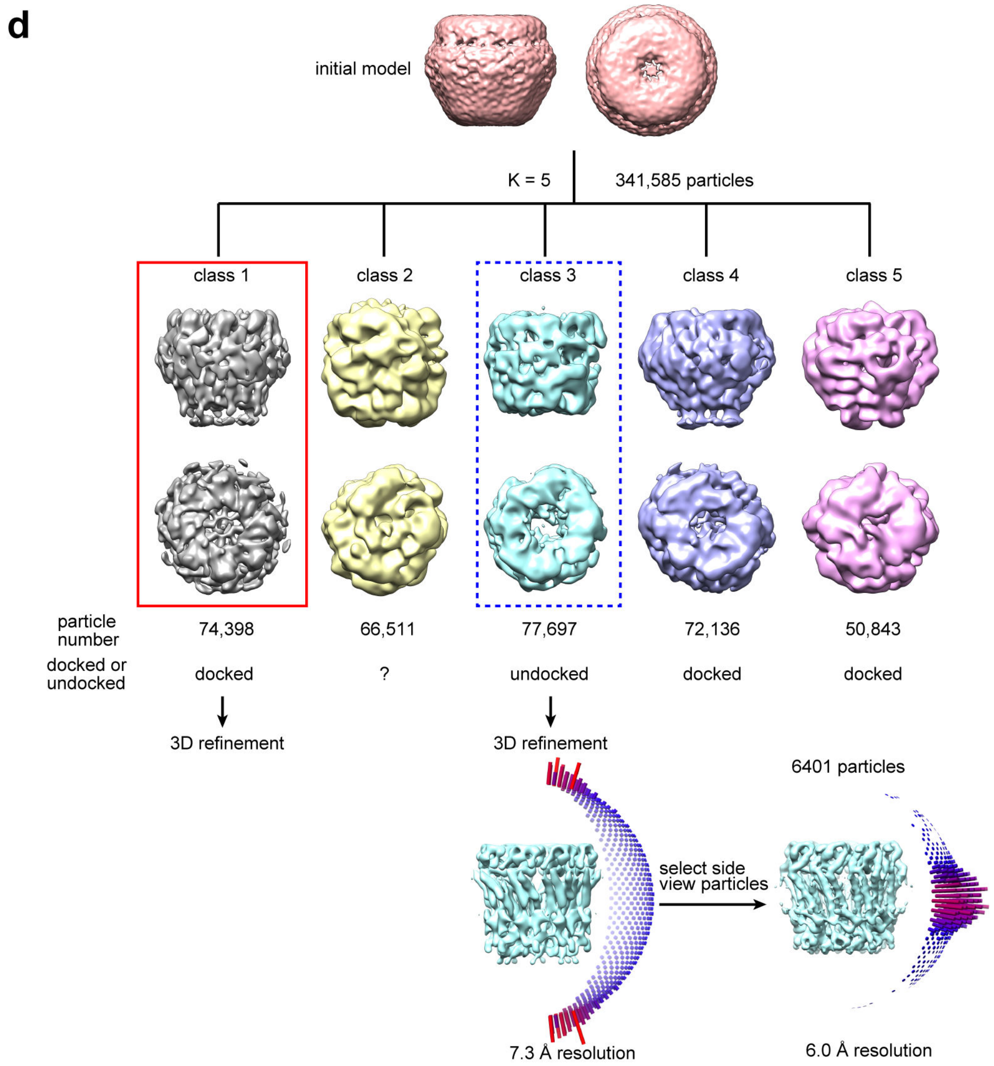

Supplementary Figure 2 | Image processing of INX-6 channels. a, Boxing INX-6 particles. An original cryo-electron micrograph (left), boxed hemichannel particles (middle) and boxed gap junction channel particles (right) are shown. Box sizes are 160 × 160 pixels for hemichannels and 200 × 200 pixels for gap junction channels. White squares represent picked particles. Scale bar, 50 nm. **b,** Two-dimensional class averages of the INX-6 hemichannel processed by EMAN2. The box size of 160 × 160 pixels corresponds to a particle window size of 197 Å. The initial model of the hemichannel (right) was generated as 160 × 160 × 160 pixels from all 32 averages shown here. **c,** Two-dimensional class averages of the INX-6 gap junction channel processed by EMAN2. The box size of 200 × 200 pixels corresponds to a particle

window size of 246 Å. The initial model of the INX-6 gap junction channel is shown on the right. **d,** Three-dimensional classification of INX-6 hemichannel particles by RELION. 74,398 particles in class 1 (docked hemichannel, red box) and 77,697 particles in class 3 (undocked hemichannel, dashed blue box) were subjected to three-dimensional refinement. The class 3 particles produced a 7.3 Å resolution map, but comprised mostly top views. The side view particles were extracted by the subsequent two-dimensional classification, but those generated a three-dimensional map at only 6.0 Å resolution.

a

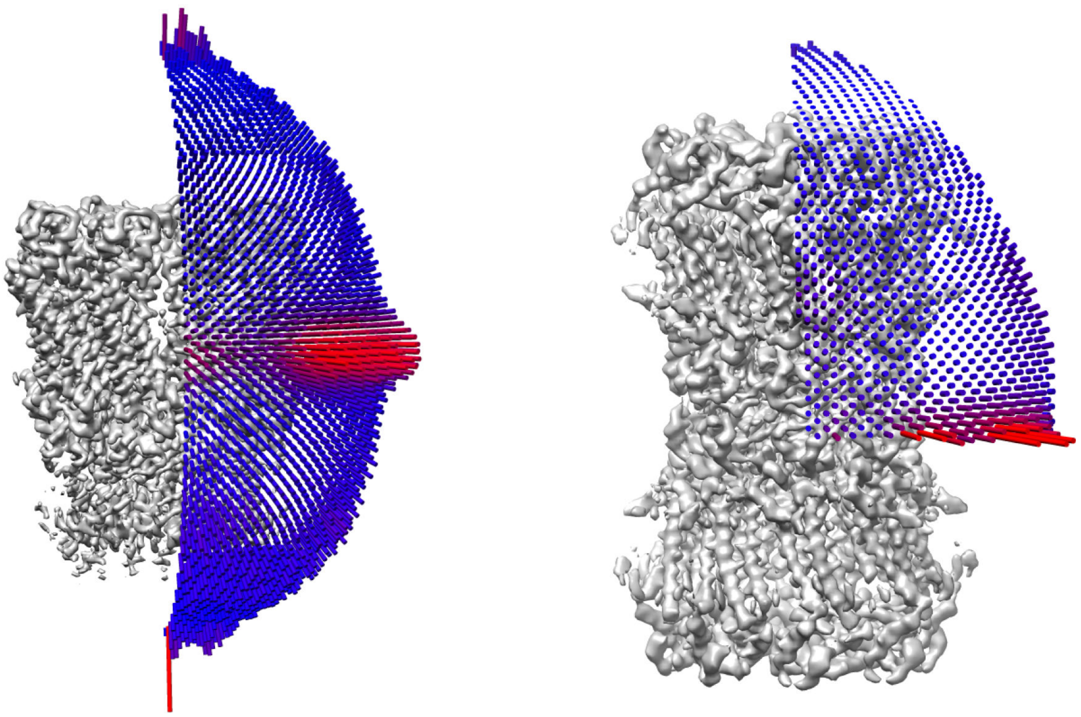

b

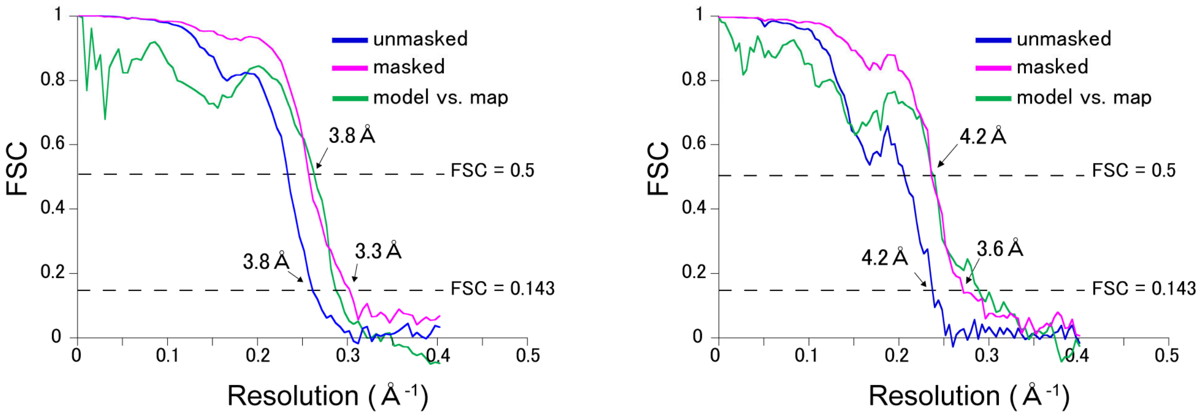

Supplementary Figure 3 | Angular distribution and resolution estimation by Fourier shell correlation (FSC) curves. a, Euler angle distribution plots of used particles for hemichannel (left) and gap junction channel (right). **b,** FSC curves of hemichannel (left) and gap junction channel (right). The curves before (blue) and after (magenta) the post-processing along with B-factor sharpening in RELION are represented. FSC curves of the model versus the final map (green) are also superimposed.

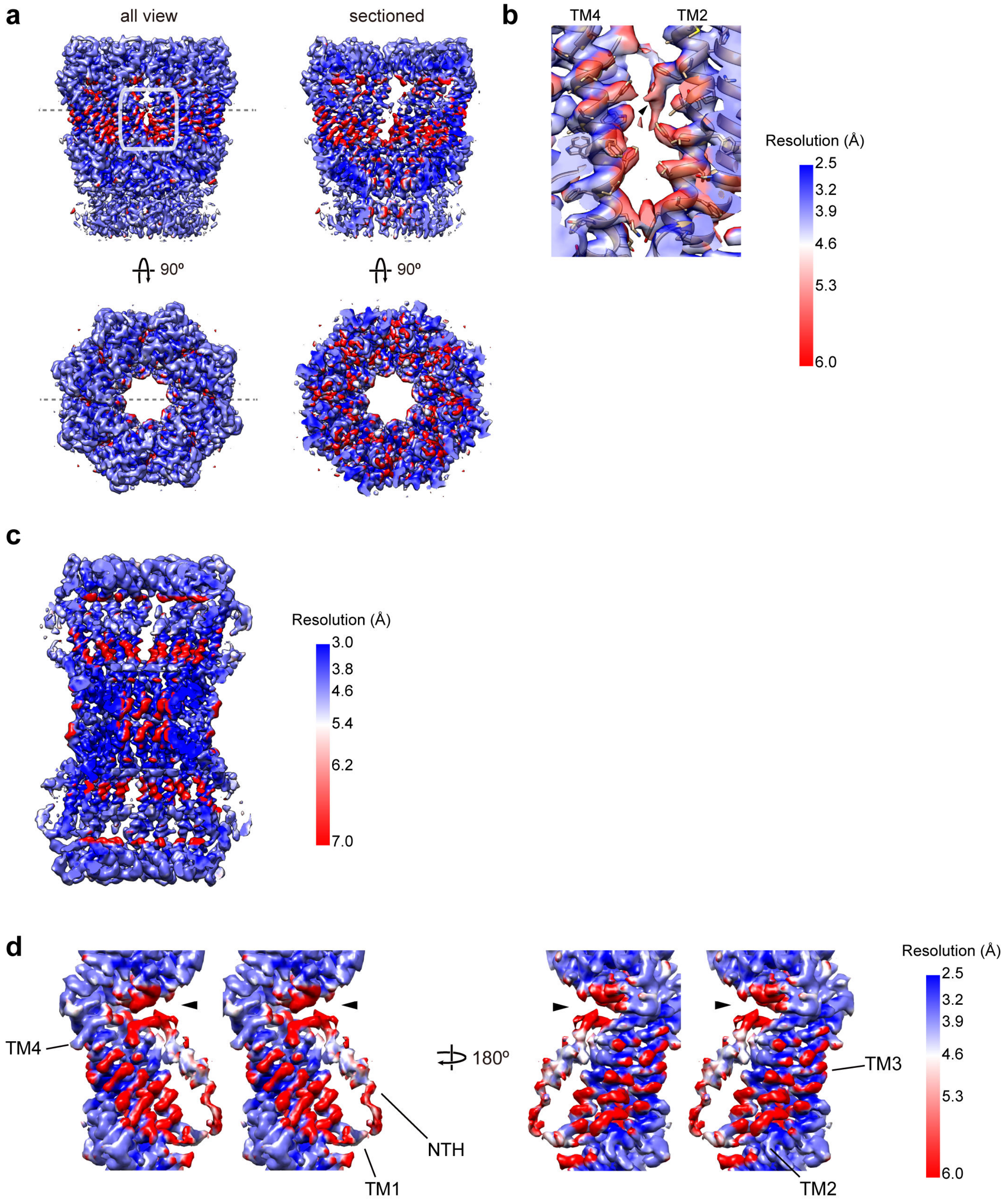

Supplementary Figure 4 | Local resolution maps of the INX-6 structures. a, Local resolution on the INX-6 hemichannel structure. Viewing all structures (left) and sectioned structures (right) in the side view

(top) and top view (bottom) are shown. The broken lines represent sectioned planes. **b,** Local resolution map around spacing between the adjacent two INX-6 subunits viewed from outside the channel, which corresponds to the white box in (a). The residues in TM4 and TM2 are depicted in stick style. The low-resolution region (red) is found at the side chains facing the space between the subunits, while the resolution of the main chain is high (blue). This is probably because the additional densities between the two subunits (arrowhead, also see Fig. 4d) are recognized as noise by RESMAP[2], resulting in underestimated resolution. The side chain densities of the transmembrane helices are sufficient for modeling (Fig. 1d). **c,** Local resolution of the INX-6 gap junction structure in a sectioned view. **d,** Stereo view of the local resolution map showing the transmembrane domain of an INX-6 monomer. The main chains of NTH and extracellular side of TM1 show higher flexibility. The position where the N- and C-terminal loops come close to each other is indicated by arrowhead.

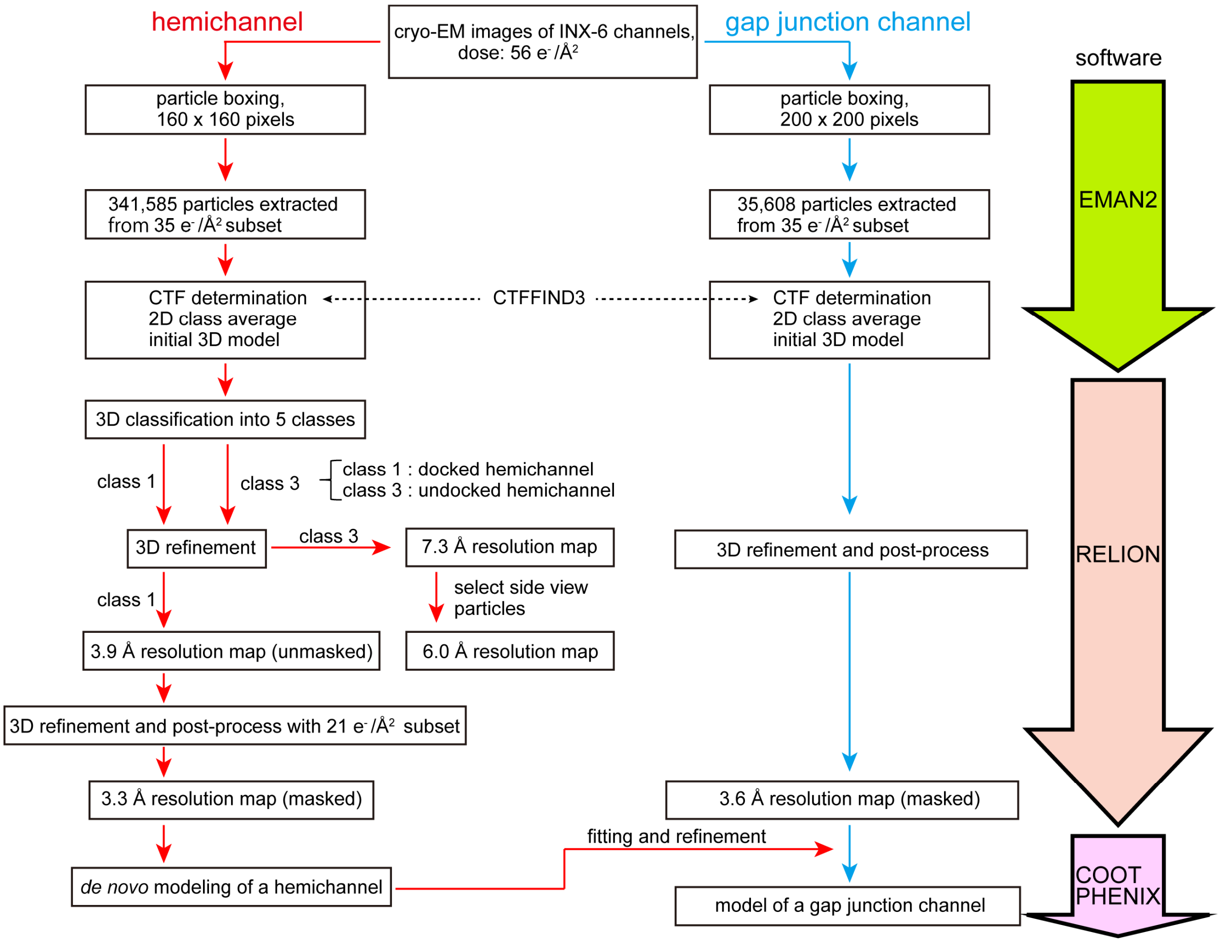

Supplementary Figure 5 | Flowchart of the image processing of INX-6 channels. The summary includes image processing steps for the hemichannel (red arrows) and gap junction channel (blue arrows). *De novo* modeling was performed with the map of a hemichannel, and the model of a gap junction channel was obtained by fitting the hemichannel model to the map of a gap junction channel followed by refinement. The processing programs we used are shown on the right.

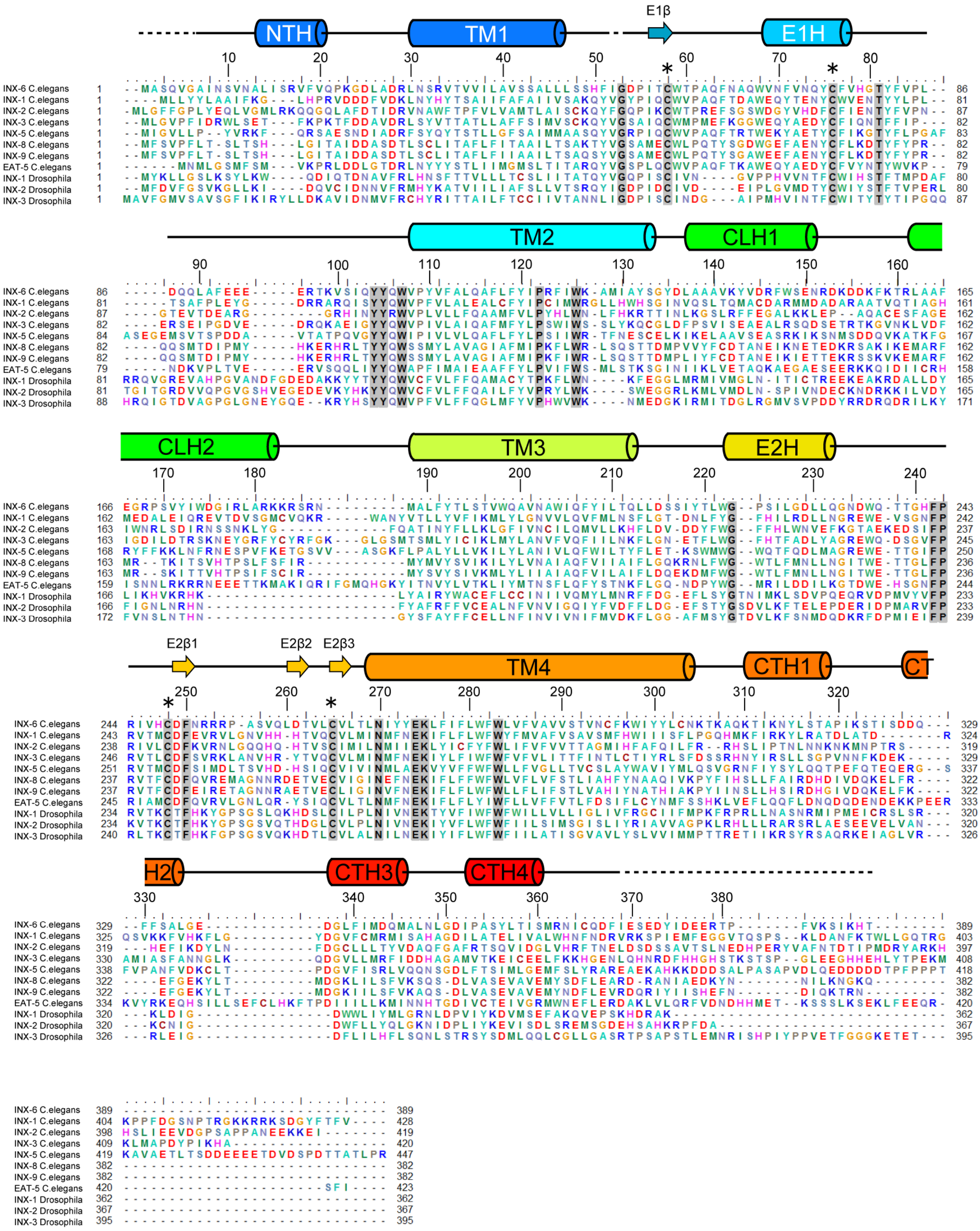

Supplementary Figure 6 | Sequence alignment of INX-6 isoforms. Selected innexin family members are aligned with their amino acid sequences by ClustalW[3]. Cartoon above the sequences represents secondary structure elements of INX-6 with the same colors as in Fig. 2, where cylinders represent helices,

arrows represent β-sheets, lines represent loops, and dashed lines represent disordered residues. Grey shaded residues are 100% conserved in this alignment. In the gap junction structure of INX-6, E2β1 is further extended and E2β3 is combined with E2β2. Strongly conserved cysteines are marked with asterisks.

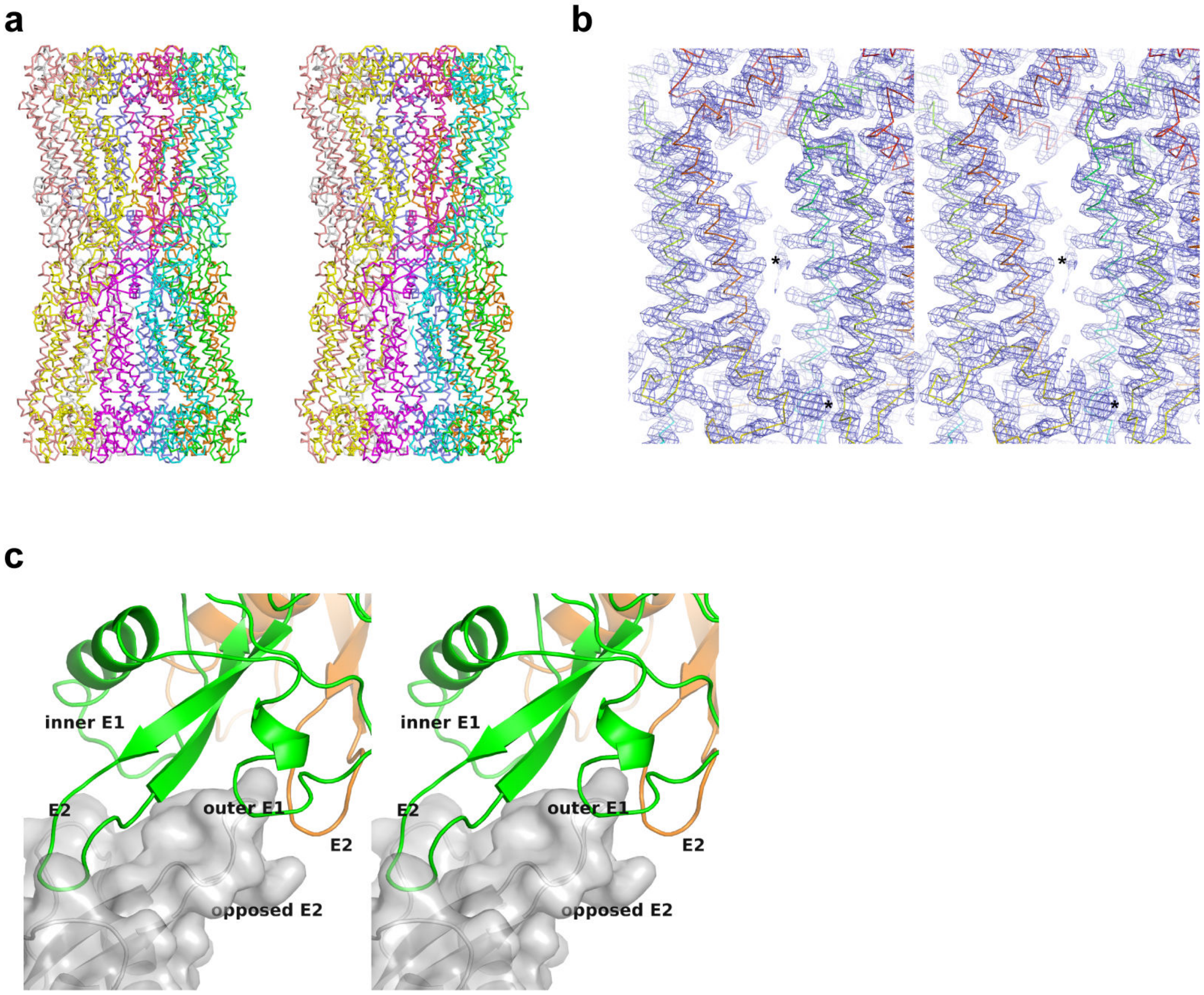

Supplementary Figure 7 | Stereo views of the INX-6 structure. a, Backbone trace of the hexadecameric gap junction structure of INX-6. **b,** Density map (blue mesh, 3σ) with the backbone of INX-6 at the spacing between the adjacent two subunits viewed from outside the channel. The densities between the subunits are marked with asterisks. The backbone color is the same as in Fig. 2. **c,** The opposed E2 β-hairpin (grey surface representation) fits into the cavity generated by the inner and outer E1 lobes and two adjacent E2s.

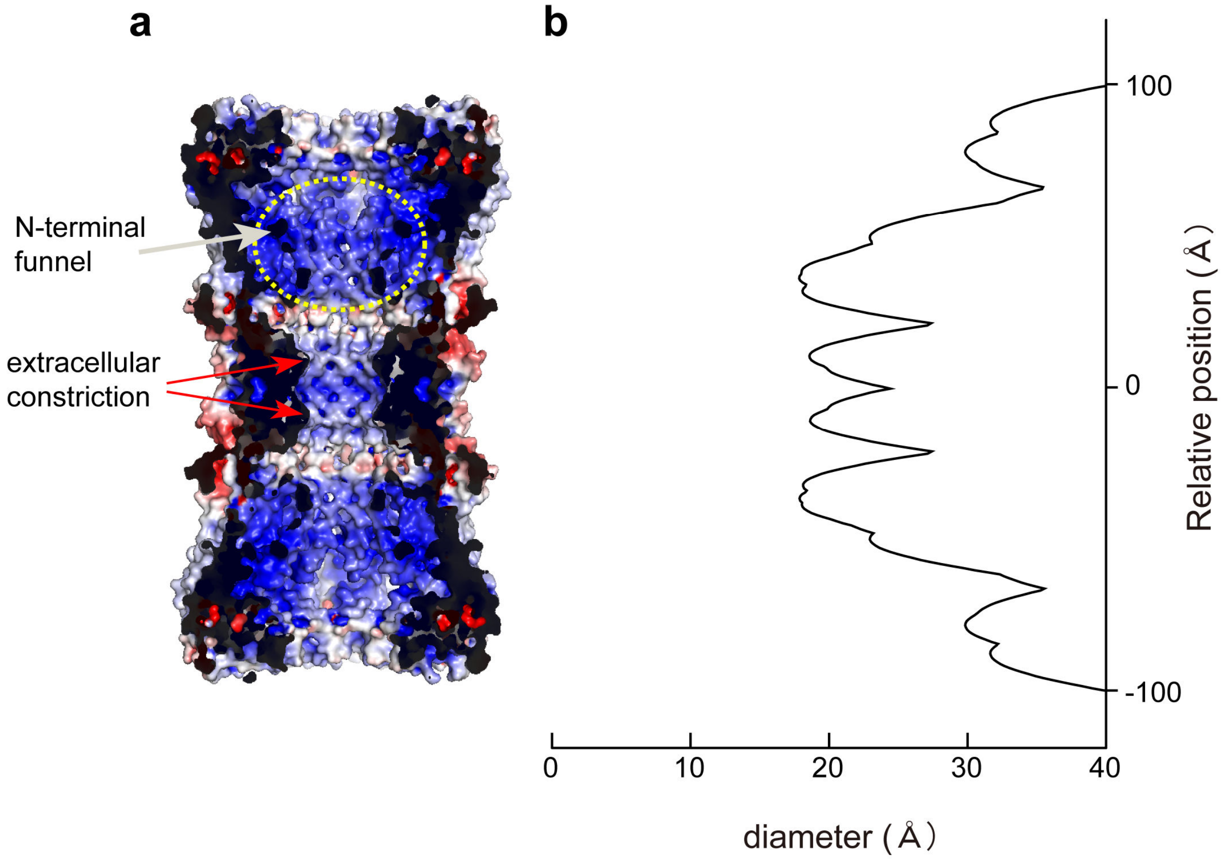

Supplementary Figure 8 | Surface potential representation and diameter of the pore pathway. a, Sectioned view of a surface representation of the INX-6 gap junction channel to show the electrostatic surface potential distribution in the pore pathway. The contour level is from –9 kT/e (red) to +9 kT/e (blue). An N-terminal funnel is indicated by a dashed yellow circle, and red arrows show the extracellular constriction sites. **b,** The pore diameter plot produced by HOLE[4]. The origin of the normal relative position is adjusted between (a) and **(**b).

		INX-6 hemichannel	INX-6 gap junction channel *
Voltage (kV)		300	300
Total image number		1053	717
Initial particle number		341,585	35,608
Final particle number		74,398	35,608 (classification not performed)
Resolution (unmasked, Å)		3.8	4.2
Resolution (masked, Å)		3.3	3.6
Defocus (μm)		0.8 - 2.4	1.2 - 2.2
Symmetry		C8	D8
Total electron dose used for refinement ($e^-/Å^2$)		35 (final refinement: 21)	35
Magnification		40,600	40,600
Pixel size (Å)		0.616	0.616
Number of frames		24	24
Exposure time (s)		7.2	7.2
B-factor ($Å^2$)		-100	-100
Ramachandran plot	Preferred	87.01%	85.84%
	Allowed	12.43%	12.75%
	Outliers	0.56%	0.94%

* Image dataset is the same as that used for the hemichannel analysis.

Supplementary Table 1 | Summary of data collection and statistics of the INX-6 structure determination

Supplementary references

1. Hauer, F. *et al.* GraDeR: membrane protein complex preparation for single particle cryo-EM. *Structure* **23**, 1769–1775 (2015).
2. Kucukelbir, A., Sigworth, F. J. & Tagare, H. D. Quantifying the local resolution of cryo-EM density maps. *Nat. Methods* **11**, 63–65 (2014).
3. Thompson, J. D., Higgins, D. G. & Gibson, T. J. CLUSTAL W: Improving the sensitivity of progressive multiple sequence alignment through sequence weighting, position-specific gap penalties and weight matrix choice. *Nucleic. Acids Res.* **22**, 4673–4680 (1994).
4. Smart, O. S., Neduvelil, J. G., Wang, X., Wallace, B. A. & Sansom, M. S. HOLE: a program for the analysis of the pore dimensions of ion channel structural models. *J. Mol. Graphics* **14**, 354–360 (1996).
